# A Systematic Review of Computational Drug Discovery, Development, and Repurposing for Ebola Virus Disease Treatment

**DOI:** 10.3390/molecules22101777

**Published:** 2017-10-20

**Authors:** James Schuler, Matthew L. Hudson, Diane Schwartz, Ram Samudrala

**Affiliations:** Department of Biomedical Informatics, Jacobs School of Medicine and Biomedical Sciences, University at Buffalo, The State University of New York, Buffalo, NY 14203, USA; jcschule@buffalo.edu (J.S.); mlhudson@buffalo.edu (M.L.H.); digs@buffalo.edu (D.S.)

**Keywords:** Ebola virus, Ebola virus disease, Ebola virus disease treatment, drug repurposing, drug repositioning, computational biology, computational pharmacology, multitargeting, polypharmacology, systematic review

## Abstract

Ebola virus disease (EVD) is a deadly global public health threat, with no currently approved treatments. Traditional drug discovery and development is too expensive and inefficient to react quickly to the threat. We review published research studies that utilize computational approaches to find or develop drugs that target the Ebola virus and synthesize its results. A variety of hypothesized and/or novel treatments are reported to have potential anti-Ebola activity. Approaches that utilize multi-targeting/polypharmacology have the most promise in treating EVD.

## 1. Introduction

### 1.1. Rationale

Ebola virus disease (EVD) is a persistent epidemic and pandemic threat with no satisfactory treatment regimen. The 2014 West Africa Ebola outbreak resulted in more than 28,000 cases leading to over 11,000 deaths, including several cases in seven countries beyond the region of West Africa [1]. This was the largest and deadliest Ebola outbreak in history and it highlighted the catastrophic potential of this emerging public health threat. Since this larger epidemic, more people have died from smaller outbreaks, most recently from May to July 2017 in the Democratic Republic of the Congo [2,3]. There are no drugs approved for the treatment of EVD and standard of care therapy remains supportive, with some clinical trials and research suggesting the use of ZMapp and hyperimmune globulin as treatments [4,5]. Novel drug discovery and development can take ten to fifteen years [6] and cost $1.5 billion per successful drug [7]. Drug development to combat Ebola virus infections can be especially problematic due to the necessity of biosafety level four (BSL-4) facilities needed to conduct preclinical studies of the Ebola virus [8].

The convergence of several technological trends over the last few decades has been a tremendous boon to computational science and informatics: Moore’s law, the Internet, exponential improvements in gene sequencing technologies, vast improvement in atomic structure elucidation techniques, tremendous population of nucleic acid and protein structure databases, and proliferation of open source scientific software each enable computation-centric research. Both computational and non-computational methods to develop treatments for EVD and other diseases or indications can engender novel drug discovery, drug development, or repurposing. Drug repurposing (repositioning) entails finding new indications for old (previously approved) drugs [9,10,11] and has many potential benefits over traditional drug discovery and development [12]. Computational approaches to drug development can provide a countervailing force against endemic burdens in traditional drug development.

Methodological aspects of computational drug discovery, development, and repurposing research have been investigated and reviewed thoroughly [13,14,15,16,17,18,19,20]. There are a variety of computational methods available to study the relationship between therapeutics, interactions, and diseases. Many of these methodologies are predicated on biological structure and the chemical influence of a structure acting within a biological system. Generally these methods are divided into structure-based and ligand-based methods [13,21]. Structure-based computer-aided drug design techniques are grounded in the understanding that structure determines function. Thus, an innate knowledge of the structure of a protein, compound, or nucleic acid is assumed to provide a guide on how interactions may occur to inhibit or augment some disease causing entity. X-ray diffraction [22], nuclear magnetic resonance (NMR) [23], and other methods provide the best starting point for use of a protein structure in subsequent analyses, as they represent the closest model of reality. Protein structure modeling, whether template-based (homology/comparative) or template free (ab initio) [24,25], is a highly utilized technique in cases where solved structures by these aforementioned techniques are unavailable, as in the case of gene products from different Ebola viral strains. Binding site prediction tools exist to locate optimal docking positions of ligands on proteins [26]. If an accurate and precise model of a ligand is similarly constructed, virtual docking and molecular dynamics simulations model the interactions between the two entities by approximating the energy of interaction in an attempt to find a minima [27]. Certain resultant poses may suggest that the binding of a ligand will inhibit the function of a protein, and thus result in a therapeutic drug. When working with data about proteins, traditional bioinformatics methods rooted in nucleic acid based sequence investigation can aid analysis. For example, careful study of the sequence of a protein encoding gene may lead to identification of further drug targets and help with their validation [28]. Additionally, high dimensional genomic data can aid in finding connections of cellular entities, including potential drug targets, further facilitating drug discovery [29].

Using only data on compound/drug chemical structure (ligand-based computer-aided drug design) one can perform pharmacophore modeling [30,31], determine quantitative structure-activity relationships (QSAR) [32,33], or calculate fingerprint based similarity metrics [27] . In pharmacophore modeling, common features of active compounds are analyzed and used to guide a search for novel therapeutics sharing similar characteristics. QSAR models the relationship between chemical characteristics and biological activity. Substructure or path based fingerprint methods quantify compound similarity to infer similar properties. The output of any method is an understanding of the nature of similarity between chemicals, often quantified as a real value score. Such a score can be used to rank potential of a compound to be used for the same indication as another [34,35]. Various visualization techniques can allow an expert investigator to examine features of both compounds and proteins, known or modeled interactions, and interaction networks [36]. Statistical and machine learning tools can help one find actionable meaning or discover nonobvious or previously unknown relationships, making them useful tools for drug discovery [37].

Computational research can utilize vast databases of compounds and biological structures and produce results quickly. The costs are minimal, humans are rarely at risk, and biosafety facilities are not required. Results highlighting the efficacy of this type of work are numerous [38,39]. One additional positive aspect of computational research is the potential ease of performing multitarget based experiments [40,41]. The function (pharmacodynamics) and efficacy (pharmacokinetics), i.e., absorption, distribution, metabolism, and excretion (ADME) of a drug involves interactions with multiple biological systems [42,43]. All drugs interact with multiple targets in the body  [44,45,46], as evidenced by the ubiquitous presence of side effects [47,48]. Therefore, there is utility in understanding the entirety of the effect of a compound holistically. Multitargeting approaches (targeting several biological entities with a single drug) are beginning to supplant traditional single target approaches (one target, one drug) [42,43,49,50,51,52,53,54,55,56]. For instance, specific tyrosine kinase receptor inhibitors such as imatinib have been shown to bind to multiple targets [54,57]. Recognizing the reality of the multitarget effects of a drug has enabled higher efficacy in treatments of infectious and neoplastic disease, allowing the possibility of evading mutation driven resistance and lower doses of individual components of drug cocktails [57,58,59,60,61,62,63,64,65]. Importantly, this paradigm has been used in the design of drugs used to target viruses [40,65,66,67], indicating its potential in the development of multitarget drugs for the treatment of EVD. Computational drug discovery research can facilitate the study of the interactome of proteins as targets of small molecule therapeutics and improve the rate of successful drug discovery, development, and repurposing. All computational work is inherently an attempt to model the real world. There are large uncertainties which come with the varying methods and approaches to computational drug discovery, development, and repurposing [21]. For this reason, purely computational work requires validation by in vivo, in vitro, or clinical studies for its results to be utilized in the real world and have a lasting impact [11,58,68,69,70,71]. There are several studies whose goal is the elucidation and development of potential treatments for EVD in preclinical and clinical studies. Many of these published results are reviewed in detail elsewhere [72,73]. Here we present a systematic review of the computational approaches which have been used to research potential therapeutics to treat EVD, with an emphasis on multitarget approaches.

### 1.2. Objectives

We aim to enumerate and highlight computational research that identify potential small molecules and biologics as drug candidates for the treatment of EVD. Focusing on commonalities in the various research studies will yield the most confidence in potential therapies. The analysis of these potential drug candidates has lead researchers to conclude that they are generally efficacious, safe, and cost effective in treating EVD. The methods and results of these computational studies are assessed on their scientific merit (especially with regard to validation studies), feasibility of development of novel therapeutic agents, and multitargeting.

## 2. Results

### 2.1. Study Selection

**Figure 1:**
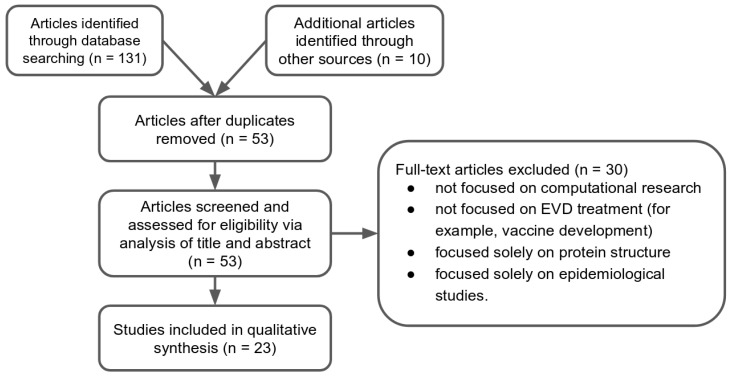
Flow diagram of the study identification process. A total of 141 publications from our initial queries were winnowed down to 23 for further consideration.

Computation-centric research is a relatively new paradigm. Similarly, Ebola virus is a relatively new, recently-emerging, global public health threat. Thus, computational research in the field of EVD treatments is limited. For instance, using the literal search term ‘“ebola” “computational” “drug”’ in a PubMed search returned only 23 results, eighteen of which were excluded by our eligibility criteria (Section 4.2). Additional queries made using both PubMed and Google Scholar were similarly winnowed down from 141 to 23 studies (Figure 1 and Section 4.2, Section 4.3, Section 4.4, Section 4.5).

### 2.2. Study Characteristics

The twenty three studies reviewed are classified according to methodologies employed: Nineteen relied on some form of virtual molecular docking, including five that integrated wet bench work. Several utilized ligand structure or pharmacophore methods and subsequent machine learning tools. Five relied on the use of a multitargeting approach (sometimes with molecular docking) and one relied on the use of DNA based sequence analysis methods. Some utilized an ensemble of methods, such as molecular docking and molecular dynamics, or virtual screening runs with candidate refinement based on criteria such as pharmacokinetic or pharmacodynamic properties.

We broadly categorize the methods used in each study into five classes:

1. Physics, dynamics, and electrostatic methods.
2. Structure activity relationships and pharmacoanalysis.
3. Bioinformatics and knowledge based methods.
4. Statistical and machine learning methods.
5. Visualization, ranking, and custom assessment.

There is some degree of subjectivity and ambiguity in this classification scheme. For example, docking can sometimes be combined with dynamics or knowledge-based scoring functions. Also, some terminology such as “virtual screening” or “structure activity relationship” are used in a broad range of contexts; a rigorously defined standard terminology or principled ontology would be helpful for future reviews of computational studies [10,74]. In the absence of a standard terminology or ontology to guide this discussion, here we loosely outline the membership of each category.

The methods of the individual published findings are categorized as follows: **physics, dynamics,** and electrostatic methods including energy minimization, geometry optimization, virtual molecular docking, molecular dynamics simulations, and computational alanine scanning; **structure activity relationship and pharmacoanalysis methods** including compound similarity methods, template-based modeling, RMSD analysis, structural fingerprinting, pharmacophore analysis, pharmacokinetics, and pharmacodynamics; **bioinformatics and knowledge based methods** including sequence alignments and analysis, residue importance prediction, and evolutionary inference; **statistical and machine learning methods** including principal component analysis, interactome similarity analysis, support vector machines (SVMs), Bayesian networks, and neural networks; and  **visualization, ranking, and custom assessment methods** including interactome signature ranking, assay integration, and visual inspection and analysis.

The studies reviewed rely on a diverse set of software tools and web servers in their approach. The most commonly used software and web servers represented in the reviewed studies include AutoDock 4 [75], the BLAST suite [76], CHARMM [77], DiscoveryStudio [78], UCSF Chimera [79], AutoDock Vina [80], GROMACS [81], MODELLER [82], Molsoft Software [83], Protox [84], and RAMPAGE [85]. There are at least several dozen distinct software tools, servers, and frameworks explicitly reported. Generally, authors report computational tools directly employed in the investigation (such as bioinformatic web servers, molecular dynamics suites, and visualization programs) and do not explicitly report indirectly employed computational tools (such as scripting languages, server operating systems, and cluster management engines). Similarly, authors often report parameters or resources for directly employed computational tools (such as docking search spaces or dynamics force fields) and not indirect parameters or resources (such as random seeds or RAM allocation). For absolute reproducibility of these studies, details of the entire software stack and hardware architecture used may be necessary.

The studies generally focus on finding inhibitors of specific Ebola virus proteins, and cover every one encoded by the Ebola virus genome: VP24, VP30, VP35, VP40, Glycoprotein (GP), Nucleoprotein, and RNA-Dependent RNA Polymerase (L) [86]. The most commonly targeted proteins were VP35 and VP40, followed by VP24 and “multiple/all proteins”.

Assorted commercial and public databases of proteins and compounds were used as sources of structures when studies required such information. The Protein Data Bank [87] and Uniprot [88] are two notable resources for investigations related to protein structure. Protein structures were downloaded from a database or, as in at least four publications, were produced using template-based modeling and in three of those, validated using RAMPAGE [85]. In keeping track of specific Ebola virus strains used in experiments, the National Center for Biotechnology Information is a valuable resource  [86,89]. GenBank accession numbers were also used to track the genetic determinants of protein structure. A multitude of databases of compounds were used as sources of chemical structures in one or more of the highlighted studies, including ZINC [90], DrugBank [91], PubChem [92], the Traditional Chinese Medicine Database (TCMD) [93], and the miRBase [94] (for the investigation of miRNA). As is the case with respect to proteins, a careful annotation of exact chemical structures enables computational work to be more easily replicated and results more meaningful.

**Figure 2:**
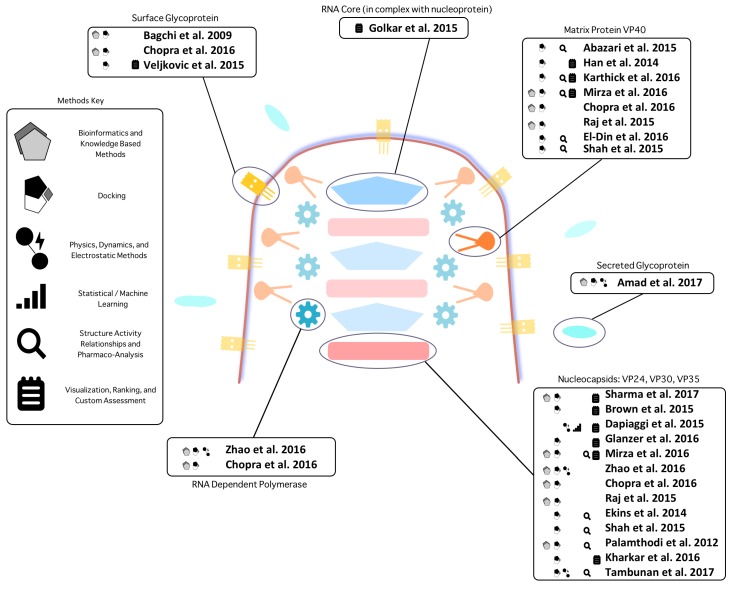
Illustration of Ebola targets investigated in the studies reviewed. The target scope of each study and broad classification of the investigatory approaches is shown. We broadly categorize distinct methods of studies into five classes: (1) **Bioinformatics and knowledge based methods** including sequence alignment and analysis, residue importance prediction, evolutionary inference, template-based modeling, and binding site prediction; (2) **Physics, dynamics, and electrostatic methods** including energy minimization, geometry optimization, molecular docking, molecular dynamics, and computational alanine scanning. (Molecular docking is the most popular methodology and is therefore distinguished separately); (3) **Structure activity relationship and pharmacoanalysis methods** including compound similarity methods, RMSD analysis, structural fingerprinting, pharmacophore analysis, pharmacokinetics, and pharmacodynamics; (4) **Statistical and machine learning methods** including principal component analysis, interactome similarity analysis, SVM models, Bayesian network models, and neural network models; (5) **Visualization, Ranking, and Custom Assessment methods** including interactome signature ranking, assay integration, and visual inspection and analysis. Target coverage is weighted towards VP24, VP30, VP35, and VP40; likely due to a greater degree of structural knowledge for these targets and that the authors tend to believe that these proteins are most important for virulence. Others often use structure prediction methods and other techniques to investigate unsolved targets. A diverse array of computational methods have been applied to find treatments against Ebola virus, and it is likely that some of them may yield effective treatments, with multitarget approaches, represented in multiple target boxes, indicating the most promise.

The scope of the current set of published approaches is presented in Supplementary Materials Table S1. Along with delineating the general computational methods used, we show the set of databases, software packages and algorithms, compounds, structures, and conclusions of each study. Figure 2 illustrates the scope of the research studies and the methodological categories they fall under. Table 1, Table 2, Table 3, Table 4 and Table 5 show the metadata of each included study, an overview of multitargeting and non-computational approaches, a selection of the top predicted compounds, a selection of software used, and the frequency and type of software used.

## 3. Discussion

### 3.1. Synthesis of Results

The salient points of all studies are included in Supplementary Table S1. A multitude of methods, software, compounds, biologics, and protein structures (modeled or solved) have been used to computationally predict therapeutics for the treatment of EVD. The studies mentioned here are of highly variable quality and scientific merit. Several are not reproducible given the limited presentation of details, an issue that hinders the efficient and reliable use of results from scientific studies and its translation. This is particularly critical for EVD due to the speed at which the outbreaks happen and the lack of any treatment [119]. As an example, Brown et al. [97] report excellent experiments and results, including computational experimentation, structural contributions to the Protein Data Bank (PDB) [87] and subsequent validation attempts with non-computational techniques at discovering inhibitors of VP35. Their top inhibitors, however, are reported in the PDB under the ligand section of the submission information and in the publication under names that are difficult to search for, which makes it difficult to recapitulate or expand on the results in the context of EVD drug repositioning. Ideally, all the studies reviewed here would clearly identify and fully report their results (such as compound names and characteristics) in their publications.

The 2014 West African Ebola virus outbreak was characterized by a rapidly mutating and highly genetically variable agent [89]. Differences in the genetics of a virus affect protein structure and function. Similarly, changes in structure may affect binding and inhibition by compounds. Therefore, it is critical for computational studies searching for modulators of protein function to identify the exact strain of virus and/or protein structure(s) used. Additionally, identification and enumeration of compounds, drugs, or chemicals enables potential replication of computational research. Computational research allows one to study many compounds and proteins with relatively little effort in scaling up. Treatments for diseases have been discovered serendipitously from a variety of sources [120]. The highly diverse compound set represented by the databases used gives a great opportunity to discover some regimen which may work to effectively treat EVD. The variance in target coverage of each study, the diversity of the computational methodologies implemented, and the large set of putative compounds reported allows significant room for follow on studies which integrate the most promising approaches and putative candidate compounds.

As highlighted in Table 3, a variety of compounds and biologics were reported as efficacious candidates against EVD as determined by computational experiments or hybrid wet laboratory studies. Data on compounds and drugs are large, heterogeneous, and complex, and thus computational research can be highly varied and lack standardization [68,121,122]. Compound sets used in different studies are rarely the same, and thus conclusions on potential treatments of EVD are vastly different. Many mechanisms of drugs are represented in the results above. This can be viewed positively, in that it highlights one of the benefits of computational research: the ease of screening and studying large and hetereogeneous data sets in the search for treatments, and not being too focused and missing out on potentially groundbreaking discoveries. Based solely on these computational studies, no single class of compounds from the collective set of publications stand out; however, several results from individual studies are promising.

As noted previously, a multitarget approach to drug therapy will produce results with a better chance of avoiding polypharmacy and evading mutation driven resistance [57,58,59,60,61,62,63,64,65]. Several of the reviewed studies utilized a multitarget approach in their search for treatments against EVD and are thus reviewed in more depth here.

In their recently published article, Raj and Varadwaj [108] utilize a virtual screening pipeline to identify flavanoids as inhibitors of the Ebola virus proteins VP40, VP35, VP24, and VP30. Protein structures were obtained from the PDB and active sites were predicted using SiteMap from the Schrodinger suite of software [123]. A three-tiered virtual screening approach to dock flavanoids from the Timtec compound library using the Glide docking program was performed by the authors. Flavanoids with the lowest energy of docking and most drug like properties were reported as top results. Gossypetin (Timtec ST059622) and Taxifolin (Timtec ST060285) are reported as having strong docking and higher inhibitotry potential against the four Ebola virus proteins than the best developed drug and gold standard, BCX4330 [124]. Therefore, one may conclude that these flavanoids are potentially useful as a multitarget treatment option in the fight against EVD.

Similarly, while not tackling the entirety of the Ebola virus genome as potential drug targets, Mizra et al. [98] take an integrated computational approach to target VP35 and VP40 with a library of over 145,000 natural compounds, phytochemicals, and flavanoids. This library was first screened for drug-like properties. Next, those compounds with desirable properties were then subjected to docking experiments using AutoDock Vina involving structures of VP35 and VP40, whose target binding sites had been predicted using the Computed Atlas of Surface Topography of proteins (CASTp). Ninety-one compounds were identified as binding with high affinity to both proteins and thus may act as multitarget treatments of EVD. Several of the top compounds are listed in Table 3 of their publication, with both a commercial name (for example, “Timtec-ST45161107”) and an IUPAC name (“2-Oxo-*N*-(2-4-((2-oxo-2*H*-chromen-3-yl)carbonyl)-1-piperazinylethyl)-2*H*-chromene-3-carboxamide”). More details including specific binding site amino acid interactions and calculated binding energies are reported.

Shah et al. [111] used virtual screening to expand on a set of compounds which had been experimentally confirmed by Kouznetsova et al. [125] to block entry into cells of Ebola virus-like peptides. The experimental compounds and their pharmacophore and structural analogues were docked to VP24, VP35, and VP40 using AutoDock Vina. Binding efficacy and physiochemical and absorption, distribution, metabolism, excretion, and toxicity (ADMET) properties of top compounds are reported. The authors predict deslanoside, digoxin, and vinorelbine, as well as several unnamed, analogous compounds from the ZINC database as effective inhibitors of the Ebola virus based on their multiple protein binding affinities and properties.

Zhao et al. “developed a structural systems pharmacology approach, to identify drug-target interactions on a proteome scale by integrating proteome-wide ligand binding site comparison, protein-ligand docking, and molecular dynamics (MD) simulation with systems biology modeling,” and applied their strategy to find FDA approved and experimental drugs which demonstrated potential to inhibit Ebola virus proteins [12]. By focusing their proteome-wide binding site comparison and protein-ligand docking procedure on two proteins critical to the Ebola virus life cycle, RNA polymerase and VP24, the authors report indinavir and sinefungin as having the highest potential of FDA approved drugs to treat EVD. One drawback of this otherwise scientifically meritorious study is a lack of validation or a comparison to wet lab studies.

Chopra et al. also recognized the importance of multitargeting. The authors’ CANDO platform used “computational screening to assess multitarget binding and inhibition”, relying on the interaction signature of a compound with “a library of protein structures that are considered representative of the (current) structural universe, compared with how that individual compound interacts with a specific protein” [101]. A highlight of this work is the corroboration of computationally derived therapeutic candidates with experimental studies. The authors highlight compounds which are predicted by the CANDO platform and which have been experimentally shown to inhibit the Ebola virus by Johsanen et al. [126] and block the entry of Ebola virus-like particles into cells by Kouznetsova et al. [125]. This study’s top candidates to target EVD which have preclinical corroboration include niclosamide, sertraline, clomifene, mebendazole, deslanoside, and digoxin. Several of these compounds, such as deslanoside and digoxin, were also listed by Shah et al. [111] as top potential treatments of Ebola, marking an instance of concordance among experimental and several computational studies. Several of these computational studies utilized a multitarget approach and a library of FDA approved drugs, thereby enabling immediate repurposing (“off label use”) and minimizing the need for phase 1 and phase 2 trials. Top candidates from Chopra et al. [101] without validation include enfuvirtide, vancomycin, bleomycin, and octreotide.

### 3.2. Limitations

We considered only articles published in English. One seemingly apparent limitation is the search period (January 2010–August 2017). However, to our knowledge, there are few articles related to computational drug research for EVD treatment published before January 2010. Indeed, the recent Ebola outbreak in West Africa has brought this issue to the fore and spurred a great deal of research with the latest technologies and techniques. Ideally, potential therapeutics for rare/unimportant/orphan/neglected diseases would be developed for the long term benefit of the humanity. Such an effort may appear to have a low return in the short term; however, in the long term, these infectious diseases could become widespread and engulf the planet in pandemic proportions quickly and without warning. If that were to happen, there would not be ample time to develop novel therapeutics from scratch, and thus computational research in these underserved diseases is a worthwhile endeavor.

Direct comparison of disparate therapeutic candidates is difficult due to lack of uniformity of compound libraries and limited description of compounds in some publications. For instance, some authors reported compounds being studied only as “Ligand 1”, “Ligand 2”, etc. with no other identifier (such as commercial or generic drug name) supplied. Unfortunately, this defeats the purpose of such research to disseminate promising results for use in preclinical and clinical validation studies to tackle immediate, emerging, and deadly public health threats. Listing many compounds, while understanding that many of them are not ideal as treatments in their current formulation, lends itself to serving as a starting point for further research and validation. Intense and thorough investigation of a few compounds may be due to lack of computational power, or researchers wanting to further lead compound development anticipating clinical studies in the near future, thereby maximizing likelihood of a candidate being approved for a particular indication and excluding undeveloped drug candidates.

More broadly, computational research has inherent limitations. All computational work is model based, and are an approximate representation of the real world [127]. This approximation is addressed by Chopra et al. when discussing the CANDO platform, which does not consider nucleic acid-compound interactions, post-translational modifications of proteins, or cell specific protein expression and copy number [101]. However, the relative ease and cost of performing computational research, especially when working with deadly pathogens, suggests that the research reviewed herein represents a useful and important contribution for the development of safe and efficacious treatments against EVD.

## 4. Materials and Methods

### 4.1. Protocol and Registration

There is little precedent for conducting systematic reviews of applied computational research, i.e., finding, reviewing, collating, appraising, and summarizing methods and results. Additionally, the use of computational methods to tackle the threat of global pandemics due to the spread of the Ebola virus is relatively new. During our search of published articles specifically on computational methods to find treatments for EVD, we found only a handful of systematic reviews of computational methods in general, including diverse topics such as fluid dynamics, aortic dissection [128], and malaria detection [129]. Therefore, while standards exist for the scope, structure, and methodology of traditional systematic reviews, including those in the Cochrane Database which summarize controlled healthcare studies, few exist for systematic reviews of computational research. This work, which is based on the Preferred Reporting Items for Systematic Review and Meta-analysis (PRISMA) statement [130], is an early step toward the creation of a standard review methodology of computational methods [131].

### 4.2. Eligibility Criteria

We considered published studies which utilized a computational approach to drug discovery, drug development, and drug repurposing to target the Ebola virus. Likewise, we also considered research that investigated biologics, such as certain miRNAs, as potential therapeutic candidates for Ebola viral inhibition. All publications were written in English and published between January 2010 and August 2017. Further detail on the characteristics of individual studies is covered in Section 2.2. Studies focused only on protein structure prediction or determination, i.e., not as part of a larger drug discovery effort, were excluded. Also excluded were publications on vaccine development, and those where Ebola was only mentioned in passing, or as part of a larger general study on computational methods. Combined computational and wet lab studies were considered when the computational component was essential to the research design.

### 4.3. Outcomes

Outcomes of this review include drugs/compounds reported by study authors as the top/most effective in treating EVD, scores and poses returned by molecular docking and/or dynamics simulations indicating inhibition of key proteins (host or pathogen) involved in Ebola virus virulence, common features of drugs with putative *in vivo* or in vitro activity (pharmacophore methods), overlap (coverage) of computational results sets with wet lab based methods or subsequent validation studies, potentially inhibitory miRNA candidate biologics, and use of a multitargeting approach.

### 4.4. Study Information Sources

Studies were identified and selected by searching a variety of electronic databases (including PubMed and Google Scholar), scanning reference lists, and consultation with experts in the field of proteomics-based drug repurposing.

### 4.5. Search Terms

The resources mentioned above were searched for articles relevant to this systematic review including but not limited to the following terms: “computational”, “drug”, “drug development”, “drug discovery”, “drug repurposing”, “in silico”, and “in virtuale”. All searches included the term “ebola” (i.e., logical AND operation).

### 4.6. Study Selection

Titles and abstracts of articles obtained as a result of the search were reviewed together by the two first authors. A publication was removed from further consideration if it did not meet the eligibility criteria described in Section 4.2. All subsequent studies were carefully read and discussed by the authors until a consensus was reached on appropriate characterization and a succinct explanation of the reviewed publication.

### 4.7. Data Collection Process

Information regarding compounds/drugs and biologics evaluated, proteins to which compounds were docked or compared, database sources, and software used were extracted from the reviewed studies. Also extracted were results, including the names of the top candidate therapeutics to treat EVD as identified by the authors. These were often based on some quantitative metric, such as scores reported by virtual docking software.

### 4.8. Data Items

Data was collected on proteins (PDB identifiers, Uniprot accession numbers), compounds (lists, sources of structures), Ebola strains (genetics), computing capabilities (model and characteristics of the hardware on which the computational work was done), software (specific programs and algorithms used to carry out the research design), comparison of computational work to preclinical or clinical studies, preclinical and/or clinical validation of putative therapeutic candidates, and the use of a multitargeting approach.

### 4.9. Bias in Individual Studies

PRISMA guidelines state that the risk of bias in individual studies must be assessed [130]. The notion of bias in computational drug research studies is not well established, and few tools exist to systematically assess bias. There has been some work toward describing what such bias may entail. Scannell et al. [132] argue that targeting a single molecule with a compound is a bias in and of itself. This idea, which they refer to as “basic research–brute force" bias, leads to the conclusion that virtual molecular docking experiments based on a single target, single ligand approach are inherently flawed, and a better approach is to consider several targets or ligands, i.e., a multitarget approach. The approach used to validate candidate therapeutics also presents another type of bias, since studies with wet lab validation are less represented among the ones reviewed. The elucidation of this bias is not the focus of this systematic review. As reported by Cleves et al. [133], the use and reliance on two dimensional (2D) descriptors for compound screening leads to an inductive bias which precludes research on truly novel compounds. Several of the reviewed studies rely on using 2D molecular descriptors of compounds and thus may be subject to this type of bias. Moreover, screening libraries themselves may be biased. Hert et al. [134] state screening libraries used in computational work are inherently biased to contain compounds previously known to cause biologic effects, thereby indicating a potential for lack of novelty in the entire drug development process (which in and of itself is indicative of an evolutionary bias). One proposed solution to mitigate bias in screening is the development of the Directory of Useful Decoys ( DUD) by Huang et al. [135], which would allow disparate methods (i.e., various docking methods) to be compared using a single, standard set of ligands.

## 5. Conclusions

The interpretation of our systematic review suggests the possibility of several drugs/compounds that may have therapeutic benefit against EVD, and that computational methods are useful not only to discover them, but also to elucidate their mechanisms of action and their likelihood of being efficacious and eventually gaining regulatory approval. Exploring potential drugs via computational modeling is a safe, frugal, and effective method to discover, develop, or repurpose potential treatments. The time and cost advantages over traditional methods is key when attempting to find therapeutic options for the treatment of an emerging, deadly disease with pandemic potential such as Ebola. Varying levels of rigor of this research exemplifies the need for further preclinical and clinical validation of putative therapeutic agents. Multitargeting approaches, especially those that are preclinically or clinically validated, have the best potential to be the most effective. Several of the approaches in the studies reviewed have the potential to be broadly applicable to other pathogens, outbreaks, epidemics, pandemics, and general drug discovery and development. Validation studies should be undertaken before any of these therapies can be recommended for clinical use, before the next Ebola outbreak arises.

## Figures and Tables

**Table 1:** List of the publications reviewed. For each publication, the title, author and year of publication, the journal in which it was published, the publisher, and the impact factor of the journal are given. While not the most ideal criterion, the publications are listed in order of decreasing impact factor, self reported or as reported by ResearchGate (RG) [95] (NA = not available; NF = not found). When two impact factors are reported, the lower of the two is used. Research articles without corresponding impact factors are listed at the end. This ordering is used in subsequent tables. The quality of the studies is variable, and thus their contribution to the endeavor of finding treatments for EVD may be as well.

Title	Author, Reference	Journal	Publisher	Impact Factor
Small-molecule probes targeting the viral PPxY-host Nedd4 interface block egress of a broad range of RNA viruses	Han et al., 2014 [96]	Journal of Virology	American Society for Microbiology	5.076 (self reported), 4.69 (RG)
In silico derived small molecules bind the filovirus VP35 protein and inhibit its polymerase co-factor activity	Brown et al., 2014 [97]	Journal of Molecular Biology	Elsevier	4.632 (self reported), 4.68 (RG)
Integrated computational approach for virtual hit identification against Ebola viral proteins VP35 and VP40	Mirza et al., 2016 [98]	International Journal of Molecular Sciences	MDPI	3.226 (self reported), 4.01 (RG)
Discovery of anti-Ebola drugs: a computational drug repositioning case study	Kharkar et al., 2016 [99]	RSC Advances	Royal Society of Chemistry	3.108 (self reported), 3.06 (RG)
Virtual screening of inhibitors targeting at the viral protein 40 of Ebola virus	Karthick et al., 2016 [100]	Infectious Diseases of Poverty	BioMed Central	3.181 (self reported), 2.97 (RG)
Combating Ebola with Repurposed Therapeutics using the CANDO platform	Chopra et al., 2016 [101]	Molecules	MDPI	2.861 (self reported), 2.80 (RG)
In silico study of VP35 inhibitors: from computational alanine scanning to essential dynamics	Dapiaggi et al., 2015 [102]	Molecular BioSystems	Royal Society of Chemistry	2.781 (self reported), 2.92 (RG)
Drug repurposing to target Ebola virus replication and virulence using structural systems pharmacology	Zhao et al., 2016 [12]	BMC Bioinformatics	BioMed Central	2.448 (self reported), 2.97 (RG)
In silico and in vitro methods to identify ebola virus VP35-dsRNA inhibitors	Glanzer et al., 2016 [103]	Bioorganic & Medicinal Chemistry	Elsevier	2.930 (self reported), 2.29 (RG)
Inhibition of Ebola Virus by anti-Ebola miRNAs in silico	Golkar et al., 2015 [104]	The Journal of Infection in Developing Countries	Open Learning on Enteric Pathogens	1.67 (RG)
Molecular modeling, simulation and docking study of Ebola Virus glycoprotein	Ahmad et al., 2017 [105]	Journal of Molecular Graphics and Modelling	Molecular Graphics and Modelling Society; American Chemical Society	1.12 (RG)
Molecular docking based screening of predicted potential inhibitors for VP40 from Ebola virus	Abazari et al., 2015 [106]	Bioinformation	Biomedical Informatics Publishing Group	0.80 (RG)
Molecular docking based screening of compounds against VP40 from Ebola virus	El-Din et al., 2016 [107]	Bioinformation	Biomedical Informatics Publishing Group	0.80 (RG)
Flavonoids as multi-target inhibitors for proteins associated with Ebola virus: in silico discovery using virtual screening and molecular docking studies	Raj et al., 2015 [108]	Interdisciplinary Sciences: Computational Life Sciences	Springer	0.753 (self reported), 0.64 (RG)
Pharmaco-Informatics: Homology Modeling of the Target Protein (GP1,2) for Ebola Hemorrhagic Fever and Predicting an Ayurvedic Remediation of the Disease	Bagchi et al., 2009 [109]	Journal of Proteomics & Bioinformatics	OMICS International	1.57 (self reported), 0.41 (RG)
Identification of novel Ebola virus (EBOV) VP24 inhibitor from Indonesian natural products through in silico drug design approach	Tambunan et al., 2017 [110]	AIP Conference Proceedings 10 July 2017	American Institute of Physics	0.22 (RG)
Pharmacophore based virtual screening and molecular docking studies of inherited compounds against Ebola virus receptor proteins	Shah et al., 2015 [111]	World Journal of Pharmacy and Pharmaceutical Sciences	WJPPS	6.647 (self reported), 0.19 (RG)
Machine learning models identify molecules active against the Ebola virus in vitro	Ekins et al., 2015 [112]	F1000Research	Faculty of 1000	NA [113]
A common feature pharmacophore for FDA-approved drugs inhibiting the Ebola virus	Ekins et al., 2014 [114]	F1000Research	Faculty of 1000	NA [113]
Homology modeling and docking studies of VP24 protein of Ebola virus with Oseltamivir and its derivatives	Sharma et al., 2017 [115]	Chemical Biology Letters	Integrated Science	NA
Virtual screen for repurposing approved and experimental drugs for candidate inhibitors of EBOLA virus infection	Veljkovic et al., 2015 [116]	F1000Research	Faculty of 1000	NA [113]
In silico analysis suggests repurposing of ibuprofen for prevention and treatment of EBOLA virus disease	Veljkovic et al., 2015 [117]	F1000Research	Faculty of 1000	NA [113]
Identification of Drug Lead Molecules against Ebola Virus: an In Silico Approach	Palamthodi et al., 2012 [118]	Journal of Computational Methods in Molecular Design	Scholars Research Library	NF

**Table 2:** Protocols used by the studies reviewed. The general protocol/method/pipeline, and their classification according to whether they include in vitro integration and/or multitargeting, is given for each study. We classify five studies as integrating in vitro methodology (such as assays, X-ray diffraction, or other methodology) and five studies as taking a multitargeting approach. Multitargeting approaches appear to have the best potential to be the most effective in the search for EVD treatment (particularly along with preclinical and clinical validation).

Author, Reference	Method/Protocol/Pipeline	In Vitro Integration	Multitargeting
Han et al., 2014 [96]	in vitro methods, docking, energy minimization, ranking, substructure similarity searching, statistical analysis (analysis of variance), testing in vitro	Yes	No
Brown et al., 2014 [97]	docking, energy minimization, ranking and interaction fingerprint comparison, medicinal chemistry: crystallography, compound synthesis, NMR spectroscopy, structural study, pulldown assay, mini genome study, EBOV assays	Yes	No
Mirza et al., 2016 [98]	binding site prediction, drug similarity analysis, pharmacokinetics, pharmacodynamics, energy minimization, metabolic site prediction, docking, validation	No	Yes
Kharkar et al., 2016 [99]	ligand-based virtual screening, molecular docking	No	No
Sharma et al., 2017 [115]	template-based modeling, structure prediction, energy minimization, validation, docking	No	No
Karthick et al., 2016 [100]	energy minimization, virtual screening, docking, intermolecular interaction analysis, dynamics, absorption-distribution-metabolism-excretion analysis (ADME), drug likeness analysis, toxicity prediction	No	No
Chopra et al., 2016 [101]	binding site prediction, docking, interaction signature ranking similarity	No	Yes
Dapiaggi et al., 2015 [102]	molecular dynamics, computational alanine scanning, RMSD fluctuations, bootstrap/principal component analysis	No	No
Zhao et al., 2016 [12]	binding site prediction, proteome wide binding site comparison, template-based modeling, docking, molecular dynamics	No	Yes
Glanzer et al., 2016 [103]	docking, alignment, in vitro testing, compound property analysis, residue analysis	Yes	No
Golkar et al., 2015 [104]	sequence alignment, other algorithms to predict miRNA-EBOV RNA inhibitory activity/post-transcriptional silencing	No	No
Ahmad et al., 2017 [105]	template-based modeling, structure prediction, validation, molecular dynamics, docking	No	No
Abazari et al., 2015 [106]	dynamics, docking, pharmacokinetic analysis	No	No
El-Din et al., 2016 [107]	fingerprint comparison, compound modeling, energy minimization, docking, pharmacokenetics	No	No
Raj et al., 2015 [108]	energy minimization, binding site prediction, docking, active site residue interaction analysis, absorption-distribution-metabolism-excretion-toxicity (ADMET) analysis	No	Yes
Bagchi et al., 2009 [109]	template-based modeling, structure prediction, validation, docking	No	No
Tambunan et al., 2017 [110]	dynamics/energy minimization, ADMET screening, molecular docking	No	No
Shah et al., 2015 [111]	pharmacophore modeling, docking	No	**Yes**
Ekins et al., 2014 [114]	pharmacophore modeling, docking	No	No
Ekins et al., 2015 [112]	viral pseudotype entry assay and EBOV replication assay, machine learning models (Bayesian, SVM, Recursive Partitioning forest, single tree), validation, virtual screening	Yes	No
Veljkovic et al., 2015 [116]	library curation/data mining, compound virtual screening (electron-ion interaction potential/average quasi valence number)	Yes	N/A
Veljkovic et al., 2015 [117]	compound virtual screening (electron-ion interaction potential/average quasi valence number), ligand optimization, molecular docking	No	No
Palamthodi et al., 2012 [118]	screening of lead molecules, docking, pharmacoanalysis	No	No

**Table 3:** A selection of the putative leads against Ebola reported in the studies reviewed. miRNAs (one study) and compound classes (two studies) are sometimes reported as leads. Some authors report self-labeled or unlabeled compounds. Compound identifiers are reported in a variety of different ways, including generic and commercial names, IUPAC terms, and PubChem, ZINC, PDB small molecule, Timtec, and Analyticon identifiers. A large set of putative leads is reported. Computational research can allow for researchers to investigate much larger sets of candidate compounds than traditional drug discovery methods.

Author, Year, Country	Reported Candidate Compounds and Biologics
Han et al., 2014, USA [96]	compounds ’4’ (Amb123203) and ’5’ (Amb21795397)
Brown et al., 2014, USA [97]	GA-017, GA-246, VPL-42, VPL-57, VPL-60, VPL-51, VPL-58, VPL-15, VPL-48, VPL-29
Mirza et al., 2016, Pakistan, Belgium [98]	Timtec-ST45161107, Otava_7118230235, Timtec-ST50912611, Timtec-ST50616170, Analyticon-NP-010155, Otava-0115540195, Analyticon-NP-019744 (kihadarnin A), Analyticon-NP-0005474, PubChem CID 17597017, Analyticon-NP-000375 (lactupicrin), Analyticon_NP-014205 (parfumine), Analyticon-NP-014522, Analyticon-003228 (isorutarin)
Kharkar et al., 2016, India [99]	sitaxentan, alitretinoin, ceftriaxone, acitretin, cidofovir, telmisartan, nateglinide, ceftizoxime, treprostinil, tenoxicam
Sharma et al., 2017, India [115]	ZINC_77287098 (an oseltamivir derivative)
Karthick et al., 2016, (Hong Kong) China [100]	Top results: emodin-8-beta- d-glucoside, tonkinochromane_G. Other results: neoglucobrassicin; glisoflavanone; rosmarinic_acid_ethyl_ester; 2-[(6Z.9Z_12Z)-heptadeca-6_9_12-trienyI]-6-hydroxybenzoic_acid; chrysophanol-8-beta-d-glucoside; 3_4-dihydro-3-methoxypaederoside; Melittoside; beta-methoxylforsythoside; glucobrassicin; manninotriose; d-mannitol_monohexadecanoate; 4__*O*-methyl_myricetin_3-*O*-(6-*O*-alpha-l-rhamnopyranosyl)-beta-d-glucopyranoside; (-)-epicatechin-3-O-gallate
Chopra et al., 2016, USA [101]	niclosamide, sertraline, clomifene, mebendazole, deslanoside, digoxin, raloxifene, clemastine, tamoxifen
Dapiaggi et al., 2015, Italy [102]	GA-017, GA-246, VPL-27, VPL-29, VPL-42, VPL-48, VPL-57, VPL-58, VPL-60
Zhao et al., 2016, USA [12]	Top results: indinavir, sinefungin. Other results: maraviroc, abacavir, telbivudine, cidofovir, montelukast, iloprost, salmeterol xinafoate, travoprost, latanoprost, remikiren, vitamin K1, mitoxantrone, labetalol hydrochloride, tafluprost, misoprostol, carboprost, fosinopril, Benzylpenicilloyl Polylysine, Bimatoprost, Nebivolol, valrubicin, Tamsulosin, Mycophenolate Mofetil, SAM, aza-Sadenosyl-Lmethionine, A9145C, Maraviroc, Telbivudine, Cidofovir
Glanzer et al., 2016, USA [103]	ZINC_05328460
Golkar et al., 2015, Denmark, USA [104]	hsa-miR-607, hsa-miR-5699-5p, hsa-miR-4682, hsa-miR-4692, hsa-miR-548az, hsa-miR-4526, hsa-miR-3065-5p, hsa-miR-145-3p, hsa-miR-491-3p, hsa-miR-4633-3p, hsa-miR-491-3p, hsa-miR-548-3p
Ahmad et al., 2017, Pakistan [105]	dronedarone 1D, amiodarone 2A, and other dronedarone and amiodarone derivatives
Abazari et al., 2015, Iran [106]	10 unlabeled, 4 selected as top candidates
El-Din et al., 2016, Egypt [107]	PubChem CIDs: 416,724, 374,108, 3,851,453, 256,623, 44,149,862, 254,616, 3183
Raj et al., 2015, India [108]	Gossypetin and Toxifolin (top 2). Other relevant results: ST50903219, ST50940361, ST101866, ST078351.
Bagchi et al., 2009, India [109]	andrographolide
Tambunan et al., 2017, Indonesia [110]	cycloartocarpin, letestuianin B, lissoclin A, varamine A, lissoclibadin 4, cystodytin J, (−)- *N*-methylcoclaurine, (−)-matairesinol, cardamonine, reticuline
Shah, et al., 2015, India [111]	deslanoside, digoxin, vincristine, vinorelbine, and several unnamed ZINC compounds and investigational compounds
Ekins et al., 2014, USA [114]	selective estrogen receptor modulators (SERMs) and anti-malarials
Ekins et al., 2015, USA [112]	quinacrine, pyronaridine, tilorone
Veljkovic et al., 2015, Serbia, France, USA, Canada [117]	267 approved and 382 experimental drugs. Notable classes: antimalarials and antibiotics (macrolides, pleuromutilins , aminoglycosides).
Veljkovic et al., 2015, Serbia, The Netherlands, Canada, USA [116]	ibuprofen
Palamthodi et al., 2012, India [118]	VP35 compounds: 2-(2,3-diamino-3-oxopropyl)sulfynyl acetic acid; 5-cyclohexypyridine 2-caboxylic acid; Copper carboxymethoxyananide dihydrate; 2, 3-dihydroxy-3-[(4-methylphenyl)carbamoyl]propanoic acid. VP40 compounds: 2-(1,3-benzothiazol-2-ylsulfanyl)acetate; 2-(1,8-dihydroxy-9-oxo-10h-anthracen-2yl)acetic acid; 1-[(2 *s*,4*s*,5*r*)-4-hydroxy-5-methyloxolan-2-yl]-5-methylpyrimidine,2,4 dione; 1-[(2*r*, 4*s*, 5*s*)-5-(hydroxymethyl)-4-methyloxolan-2-yl]-1,2,4-triazole-3-carboxamide.

**Table 4:** A selection of software used in the studies reviewed. Generally, authors only reported primary scientific software used. Other software, automation scripts, operating systems, cluster management software, APIs, or other elements of the studies were usually not specifically reported. Some authors reported abstract or architectural descriptions of computational pipelines or platforms. Reproducibility of future work can be enhanced by reporting details on the entire software stack, software parameters, and hardware environment. A standard protocol for reporting the computational environment is needed in order to facilitate the comparison of research.

Author, Year, Country	Selected Software, Algorithms, and Version Numbers
Han et al., 2014, USA [96]	Autodock 4.0, CHARMM (MMFF), Accelrys LigScore2
Brown et al., 2014, USA [97]	Autodock 4.0 (DOVIS PIPELINE), CHARMM35b2 (MMFF), LigScore2, REFMACS, Phenix, PRODRG2, Coot, MolProbity, GraphPad Prism
Mirza et al., 2016, Pakistan, Belgium [98]	OpenBabel, Discovery Studio, UCSF Chimera (AMBER ff12SB force field for protein energy minimization), CASTP, MUSCLE, Jalview 2.7, BLAST (PSI-BLAST), ALIGN2D, MODELLER 9.12, CHARMM22, Autodock Vina, Mcule-pipeline, PyMol, DrugScore eXtended, DUD-e, Daylight, NSCC.11, Molinspiration, OSIRIS, ADMET prediction suite (ACD/LABS), Molsoft, AdmetSAR, Aggregator Advisor, MetaPrint2D, PAINS, clustalX, ligPlots
Kharkar et al., 2016, India [99]	ROCS OpenEye Scientific Software Suite, AutoDock
Sharma et al., 2017, India [115]	Phyre2, EasyModeller4, RAMPAGE, BLASTP, YASARA, Schrodinger Suite, GLIDE, Autodock, Discovery Studio Visualizer
Karthick et al., 2016, (Hong Kong) China [100]	GROMACS (GROMOS43a1), iScreen (PLANTS), Autodock 4.2.6, Autodock Tools, AutoGrid, PEARLS, PDBsum, UCSF Chimera, GROMACS (GROMOS43a1, SPC water model), PRODRG (EWALD Algorithm, Lincs Algorithm), GROMACS_UTIL (g_rms, g_hbond), Molsoft, OSIRIS Property Explorer, Protox Web Server, PROCHECK
Chopra et al., 2016, USA [101]	COFACTOR, CANDOCK, ITASSER (HHBLITS, LOMETS, SPICKER, ModRefiner, KobaMin, et al.), ChemAxon MarvinBeans molconverter v.5.11.3, Xemistry Cactvs Chemoinformatics Toolkit, CANDO
Dapiaggi et al., 2015, Italy [102]	GROMACS (AMBER99SB-ILDN, Generalized Amber Force Field, TIP3P, LINCS algorithm, velocity rescale algorithm, Berendsen barostat, Particles Mesh Ewald algorithm), (MM/PBSA, APBS), GROMACS_utility (g_covar, g_anaeig), VMD, Naccess
Zhao et al., 2016, USA [12]	Verify3D, PROCHECK, SMAP, Modeller v9.14, I-TASSER, Autodock4, Autodock Vina, PLANTS, Surflex, AutoDockTools 4, CASTp, Xleap, ACEMD (AMBER99SB, SHAKE algorithm), Pymol, Ligplot
Glanzer et al., 2016, USA [103]	Molegro Docking, Molinspiration Property Calculation Service, Molegro Structure Protein Alignment, UCSF CHIMERA
Golkar et al., 2015, Denmark, USA [104]	Software described in a cited publication
Ahmad et al., 2017, Pakistan [105]	PREDATOR, PHD, GOR4, DPM, HNN, DSC, SIMPA96, SOPM, RONN, GLOBPLOT, DISSEMBLE, MOE, RAMPAGE, ERRATE, Expasy-ProtoParam, MUSCLE server, PSI-BLAST
Abazari et al., 2015, Iran [106]	GROMACS 4.5.4, PyRx / AutoDock Vina, FAFDrugs3, admetSAR, PROTOX
El-Din et al., 2016., Egypt [107]	Chem Sketch, Swiss PDB Viewer, Autodock4 , Auto Grid, Auto Dock hydrogen module, UCSF Chimera, PROTOX, Molsoft
Raj et al., 2015, India [108]	Protein Preparation Wizard, SiteMap, GLIDE (Receptor Grid Generation Panel), QikProp v3.9
Bagchi et al., 2009, India [109]	MODELLER, Swiss-PDBViewer, ACD/ChemSketch, RAMPAGE, ArgusLab 4.0.1, HEX_SERVER, HHpred, BLAST
Tambunan et al., 2017, Indonesia [110]	Molecular Operating Environment (MOE) 2014.09, UCSF Chimera 1.9, Vega ZZ 3.0.5, OSIRIS DataWarrior 4.2.2
Shah, et al., 2015, India [111]	Discovery Studio Visualizer 4.0, Swiss PDB viewer, Chimera, CastP server, AutoDock Vina 4.2, GEMDOCK, Molinspiration
Ekins et al., 2014, USA [114]	Discovery Studio 4.1 (CAESAR, FAST conformer generation, LibDock), CHARMM
Ekins et al., 2015, USA [112]	Discovery studio, Mobile Molecular Data Sheet, R (programming language), other software described in citation
Veljkovic et al., 2015, Serbia, France, USA, Canada [116]	Software undisclosed, showed equations for AQVN and EIIP and explained high level process, secondary goal of this publication was to establish a web server for this type of study, “ebola screen” web server ( http://www.biomedconsulting.info/ebolascreen.php)
Veljkovic et al., 2015, Serbia, The Netherlands, Canada, USA [117]	Custom software for calculating AQVN and EIIP, VEGA ZZ, MOPAC 2009, Autodock Vina
Palamthodi et al., 2012, India [118]	PYMOL, Chimera, Arguslab, AutoDock

**Table 5:** Frequency of software used in the studies reviewed. A nonexhaustive sample of the types of software and servers and their minimal frequency of use for computational drug discovery methods and techniques used in the treatment against EVD. Although researchers converge on a few popular programs for common methods like molecular docking or molecular dynamics, there exists an abundance of options for nearly every methodology.

Software	Count	Method/Technique
Autodock	7	molecular docking
UCSF CHIMERA	7	visualization and analysis suite
Discovery Studio	5	affinity, ranking, modeling, workflow tooling
Autodock Vina	5	molecular docking
BLAST Suite	4	sequence alignment
CHARMM	4	molecular dynamics, minimization, analysis
Autodock Tools	3	structure preparation utilities, workflow tooling
GROMACS	3	molecular dynamics
Modeller	3	template-based modeling
Molsoft	3	suite of bioinformatic and cheminformatic tools
PROTOX	3	toxicity prediction
RAMPAGE	3	Ramachandran plot analysis
CASTP	3	binding and active site prediction/analysis
Molinspiration	3	cheminformatic software suite
Swiss PDB Viewer	3	visualization and analysis
PyMol	3	visualization
OSIRIS	3	chemical property analysis
ACD ChemSketch	2	chemical structure modeling, property analysis, logp
admetSAR	2	cheminformatics/ADMET analysis
AutoGrid	2	support tooling for AutoDock
GLIDE	2	docking
GROMACSutils	2	support tooling for GROMACS simulations
I-TASSER	2	protein structure and function prediction
LigScore2	2	binding affinity prediction
MUSCLE	2	multiple alignment
PROCHECK	2	protein structure steriochemical quality analysis
PRODRG	2	small molecule topology generation for simulation use, energy minimization
ArgusLab 4.0.1	2	molecular modeling
MOE	2	suite of protein modeling, assessment, and analysis software
VEGA ZZ	2	molecular modeling suite
ADMET prediction suite (ACD/LABS)	1	ADMET analysis
Aggregator Advisor	1	molecular aggregation prediction for biochemical assays
ALIGN2D	1	sequence structure alignment
biomedconsulting Ebola screen server	1	AQVN and EIIP screening server for Ebola research
CANDO	1	drug discovery platform (docking, dynamics, multitargeting, and drug repurposing)
CANDOCK	1	fragment based docking with dynamics
ChemAxon MarvinBeans	1	computational chemistry suite
clustalX	1	multiple alignment
COFACTOR	1	protein functional annotation
Coot	1	visualization, modeling, analysis, validation
Daylight	1	cheminformatics, fingerprinting
DisEMBL	1	Intrinsic disorder prediction
DOVIS PIPELINE	1	virtual screening pipeline
DPM	1	promoter structure of co-regulated gene modeling
DrugScore eXtended	1	knowledge based protein-ligand complex scoring
DSC	1	protein secondary structure prediction
DUD-e	1	docking benchmarking, virtual screening support
EasyModeller4	1	interface for MODELLER with integrated analysis tooling
ERRAT	1	verifying protein structures determined by crystallography
Expasy-ProtoParam	1	protein physical and chemical analysis
FAFDrugs3	1	ADMET screening, virtual screening filtering
GLOBPLOT	1	intrinstic protein disorder, domain, and globularity prediction
GOR4	1	secondary structure prediction
GraphPad Prism	1	graphing and statistics
HEXSERVER	1	docking
Hhpred	1	protein structure and function prediction
HNN	1	secondary structure prediction
iScreen	1	docking platform
Jalview	1	multiple sequence alignment editing, visualization and analysis
Ligplot+	1	schematic diagrams of protein-ligand interactions
Mcule-pipeline	1	molecular modeling and cheminformatic screening interface for mcule database
MetaPrint2D	1	xenobiotic metabolism prediction, phase I metabolic site prediction
Mobile Molecular Data Sheet	1	mobile cheminformatics modeling and analysis
molconverter	1	utility for file format conversion
Molegro Docking	1	docking
Molegro Structure Protein Alignment	1	alignment
MolProbity	1	protein structure validation
MOPAC	1	semiempirical quantum chemistry program
Naccess	1	atomic solvent accessible area prediction
NSCC 11	1	statistical software
OpenBabel	1	computational chemistry software suite
PDBsum	1	protein visualization and analysis
PEARLS	1	energetic analysis of receptor-ligand systems
PHD	1	secondary structure prediction
Phenix	1	structure determination software for X-ray crystallography and other methods
Phyre2	1	protein fold recognition
PLANTS	1	docking
PREDATOR	1	secondary structure prediction
protein preparation wizard	1	tools for protein structure preparation for simulation
PyRx	1	virtual screening pipeline
QikProp	1	ADME screening, prediction
R	1	statistical programming language/platform
REFMACS	1	maximum likelihood refinement analysis for protein structure data
ROCS OpenEye	1	virtual screening by shape comparison tool
RONN	1	protein disorder prediction
Schrodinger Suite	1	suite of protein modeling, assessment, and analysis software
SIMPA96	1	secondary structure prediction
SiteMap	1	binding site prediction
SMAP	1	protein-ligand interaction analysis
SOPM	1	secondary structure prediction
Surflex	1	docking
Verify3D	1	structure-sequence compatibility assessment
VMD	1	visualization, analysis
Xemistry Cactvs Cheminformatics Toolkit	1	cheminformatics software suite
Xleap	1	tool that interfaces with LEAP/AMBER
YASARA	1	visualization, modeling, analysis
ACEMD	1	dynamics
Gemdock	1	docking

## Supplementary Materials

A comprehensive table that collates the data selected from each study reviewed is available online as Supplementary Table S1.

## Abbreviations

ADME	absorption distribution metabolism excretion
ADMET	absorption distribution metabolism excretion toxicity
BSL-4	biosafety level four
EBOV	Ebola virus
EVD	Ebola virus disease
NMR	Nuclear magnetic resonance
PDB	Protein Data Bank
QSAR	Quantitative structure-activity relationship
RAM	Random access memory
RG	ResearchGate
RMSD	Root mean square deviation
SVM	Support vector machine
